# IRX4204 sensitizes multiple myeloma to ferroptosis and improves lenalidomide efficacy through the HMOX1-GPX4 axis

**DOI:** 10.1038/s41598-026-42123-9

**Published:** 2026-03-17

**Authors:** Jian Wu, Zhibo Yan, Kimberly Burcher, Zhannan Han, Mikhail A. Nikiforov, Vidyasagar Vuligonda, Martin Sanders, Yubin Kang

**Affiliations:** 1 Division of Hematologic Malignancies and Cellular Therapy, Department of Medicine, Duke University Medical Center, Durham, NC 27710 USA; 2 Department of Pathology, Duke University School of Medicine, Durham, NC 27710 USA; 3 Department of Biomedical Engineering, Pratt School of Engineering, Duke University, Durham, NC 27708 USA; 4 Io Therapeutics, Inc, Spring, TX 77387 USA

**Keywords:** Cancer, Cell biology, Diseases, Drug discovery

## Abstract

Despite advances in therapy, multiple myeloma (MM) largely remains incurable, emphasizing the need for new strategies to overcome drug resistance. Ferroptosis is an iron-dependent cell death pathway that may present a therapeutic vulnerability in MM, but its transcriptional regulation remains poorly understood. Retinoid X receptors (RXRs) are ligand-activated nuclear transcription factors that regulate metabolism, redox homeostasis, and immune signaling. In this study, we demonstrate that RXR signaling regulates ferroptosis. The selective third-generation RXR agonist IRX4204 significantly increased MM cells’ susceptibility to ferroptotic stress and worked synergistically with ferroptosis inducers. Mechanistic studies showed that IRX4204 actively induces HMOX1 transcription via PPARα-RXRα binding and concurrently decreases GPX4 levels, leading to iron buildup, lipid peroxidation, and ferroptosis. Deleting HMOX1 using CRISPR abolished these effects, confirming HMOX1 as an essential effector. In vivo, IRX4204 enhanced lenalidomide’s effectiveness, reduced tumor burden, extended survival, and elevated ferroptosis markers without added toxicity. Clinically, high HMOX1 expression correlates with improved overall survival in MM patients. These findings reveal a new RXR–HMOX1–GPX4 regulatory axis, establish RXR activation as a method to boost ferroptosis sensitivity, and support combining RXR agonists with ferroptosis-based treatments in MM.

**Supplementary Information:**

The online version contains supplementary material available at 10.1038/s41598-026-42123-9.

## Introduction

Multiple myeloma (MM) is a plasma cell malignancy characterized by clonal proliferation within the bone marrow, which causes complications such as bone destruction, anemia, kidney dysfunction, and impaired immunity^1^. Despite major advances achieved through proteasome inhibitors, immunomodulatory drugs, monoclonal antibodies, and T cell-based therapies, MM remains largely incurable. Most patients ultimately relapse due to drug resistance, metabolic adaptation, and immune evasion^2^. These challenges highlight the need for new treatment approaches that exploit alternative cellular vulnerabilities in order to achieve more sustained disease control.

Retinoid X receptors (RXRs) are ligand-responsive transcription factors within the nuclear receptor superfamily and function as required heterodimeric partners for several receptors, including the peroxisome proliferator-activated receptor (PPAR) family^3–6^. By forming these permissive heterodimers, RXR signaling coordinates pathways involved in lipid metabolism, cellular responses to oxidative stress, and immune regulation. Our earlier work showed that stimulating RXR activity with the selective agonist LG100754 strengthened the antimyeloma effects of lenalidomide while improving T-cell function and maintaining systemic metabolic stability^7^. These results underscored the therapeutic value of targeting RXR in myeloma and identified RXR as an important transcriptional node connecting metabolic pathways with immune activity. However, the broader mechanisms through which RXR activation exerts anti-myeloma effects remain incompletely defined.

Ferroptosis is an iron-dependent, lipid peroxidation-driven form of regulated cell death that has emerged as a promising therapeutic target in MM^8^. We further showed that targeting serine/threonine kinase 17B (STK17B) can trigger ferroptosis and reverse treatment resistance in MM cells^8^. This iron-dependent cell death pathway is driven by the accumulation of lipid hydroperoxides in cellular membranes, resulting from impaired antioxidant defense systems, particularly the glutathione peroxidase 4 (GPX4)/glutathione axis^9^. Given that myeloma cells exhibit heightened oxidative metabolism, elevated immunoglobulin synthesis, and increased iron dependency to sustain their proliferative demands, they may be intrinsically sensitive to ferroptotic stress when antioxidant defenses are compromised.

The mechanistic intersection between RXR signaling and ferroptosis regulation has not been explored in myeloma. Nuclear receptors, including PPARα, regulate genes involved in lipid metabolism and redox homeostasis; it is plausible that activating RXR could influence ferroptosis sensitivity through transcriptional reprogramming^10^. Heme oxygenase 1 (HMOX1) is an inducible enzyme that oxidizes cellular heme to produce biliverdin, carbon monoxide (CO), and free ferrous iron, making it a strong candidate as a mediator in this process^11^. While HMOX1 is classically considered cytoprotective under oxidative stress, emerging evidence indicates that excessive HMOX1 induction can paradoxically promote cell death by increasing labile iron pools and driving lipid peroxidation^12,13^. This dual role implies that HMOX1 acts as a context-dependent modulator of ferroptosis, though its transcriptional control by RXR has not been investigated.

IRX4204 is a third-generation RXR agonist characterized by strong receptor selectivity and minimal cross-activation of retinoic acid receptors (RARs), which enhances its pharmacologic precision and safety profile^14^. Although preclinical studies have demonstrated that IRX4204 exerts potent anti-tumor and immunoregulatory effects in several cancer models, its underlying mechanisms in MM are still not well understood^15,16^. Given the established role of RXR in metabolic regulation and our previous findings linking RXR activation to enhanced anti-myeloma activity, we hypothesized that IRX4204 might modulate ferroptosis sensitivity in MM cells by regulating transcription of iron metabolism and antioxidant defense pathways. In the current study, we investigated whether RXR activation by IRX4204 could potentiate ferroptosis and thereby overcome treatment resistance. Here, we report that IRX4204 transcriptionally activates HMOX1 through PPARα: RXRα heterodimer binding to the HMOX1 promoter, leading to increased labile iron accumulation and enhanced ferroptosis sensitivity. These effects synergize with lenalidomide to improve anti-tumor efficacy in vivo without additional toxicity. Our findings establish a previously unrecognized RXR–HMOX1–GPX4 regulatory axis that connects nuclear receptor signaling to ferroptosis regulation, and provide a mechanistic rationale for combining RXR agonists with immunomodulatory drugs or ferroptosis-inducing agents in the treatment of drug-resistant MM.

## Results

### IRX4204 induces ferroptosis-dependent cell death in multiple myeloma

**Fig. 1:**
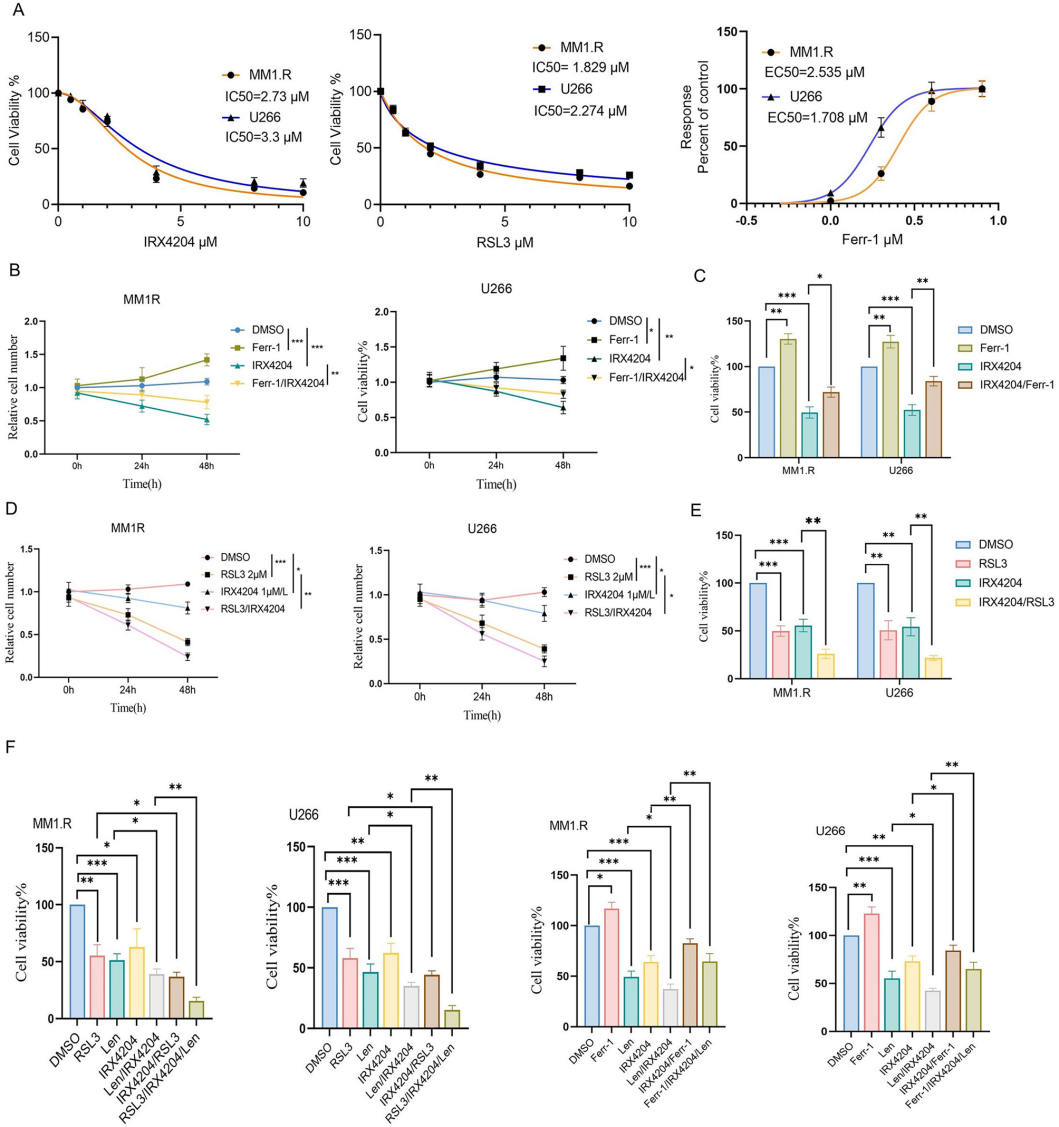
IRX4204 sensitizes multiple myeloma cells to ferroptosis in vitro. (**A**) Dose–response curves of MM1.R and U266 cells treated with IRX4204, RSL3, or Ferr-1. (B-C) Ferr-1 treatment rescued IRX4204-induced cytotoxicity in MM. MM1.R cells (left panel) and U266 cells (right panel) were treated with DMSO, Ferr-1(2 µM), IRX4204 (3 µM) or the combination of Ferr-1 and IRX4204. Cell number was measured at 0, 24, and 48 h following treatment (**B**). Cell viability was assessed at 48 h by the MTT assay (**C**). (D-E) Co-treatment with RSL3 and IRX4204 led to enhanced cytotoxicity to MM cells. MM1.R cells (left panel) and U266 cells (right panel) were treated with DMSO, RSL3 (2 µM), IRX4204 (3 µM) or the combination of RSL3 and IRX4204. Cell number was measured at 0, 24, and 48 h following treatment (**D**). Cell viability was assessed at 48 h by the MTT assay (**E**). (**F**) MM1.R cells and U266 cells were treated with DMSO, RSL3 (2 µM), Ferr-1(2 µM), IRX4204 (3 µM), lenalidomide (Len, 10 µM) or various combination for 48 h. Cell viability was measured. Statistical analysis was performed using one-way ANOVA followed by Tukey’s multiple comparison test (Fig. 1B-E). All experiments performed in triplicate; *p < 0.05, **p < 0.01, ***p < 0.001.

To determine whether RXR activation influences ferroptosis in MM, we first assessed the cytotoxic response of MM1.R, U266, and RPMI8226 MM cell lines to IRX4204, the ferroptosis inducer RSL3, and the ferroptosis inhibitor Ferrostatin-1 (Ferr-1). MM cell lines showed dose-dependent growth inhibition in response to IRX4204 and RSL3, with IC₅₀ values in the low micromolar range (Fig. 1A, Supplementary Fig. 1 A). In contrast, Ferr-1 exhibited a sigmoidal growth curve consistent with its protective activity (Fig. 1A, Supplementary Fig. 1 A).

Critically, co-administration of Ferr-1 effectively reversed IRX4204-induced cytotoxicity, restoring cell survival over 48 h (Fig. 1B-C), demonstrating that IRX4204 causes cell death specifically through ferroptosis. This ferroptosis-dependent mechanism was further confirmed by the synergistic cytotoxicity observed when IRX4204 was combined with RSL3 (Fig. 1D-E, Supplementary Fig. 1B). The combined treatment significantly reduced cell viability compared with either agent alone in both MM1.R and U266 cells, and time-course analysis confirmed a progressive decline in viable cell numbers following dual treatment (Fig. 1D-E).

To explore the therapeutic relevance of IRX4204-induced ferroptosis, we evaluated whether it could enhance lenalidomide efficacy. Consistent with our previous findings using other RXR agonists, IRX4204 significantly boosted lenalidomide-induced cytotoxicity, and the triple combination of IRX4204, RSL3, and lenalidomide produced the greatest reduction in cell viability across both cell lines (Fig. 1F). Notably, Ferr-1 partially rescued this effect, confirming that ferroptosis contributes to the increased anti-myeloma activity of the IRX4204-lenalidomide combination.

Collectively, these results demonstrate that IRX4204 induces ferroptosis-dependent cell death in MM cells and synergizes with both ferroptosis inducers and lenalidomide through this mechanism.

### IRX4204-induced ferroptosis involves suppression of antioxidant defenses and accumulation of oxidative stress

**Fig. 2:**
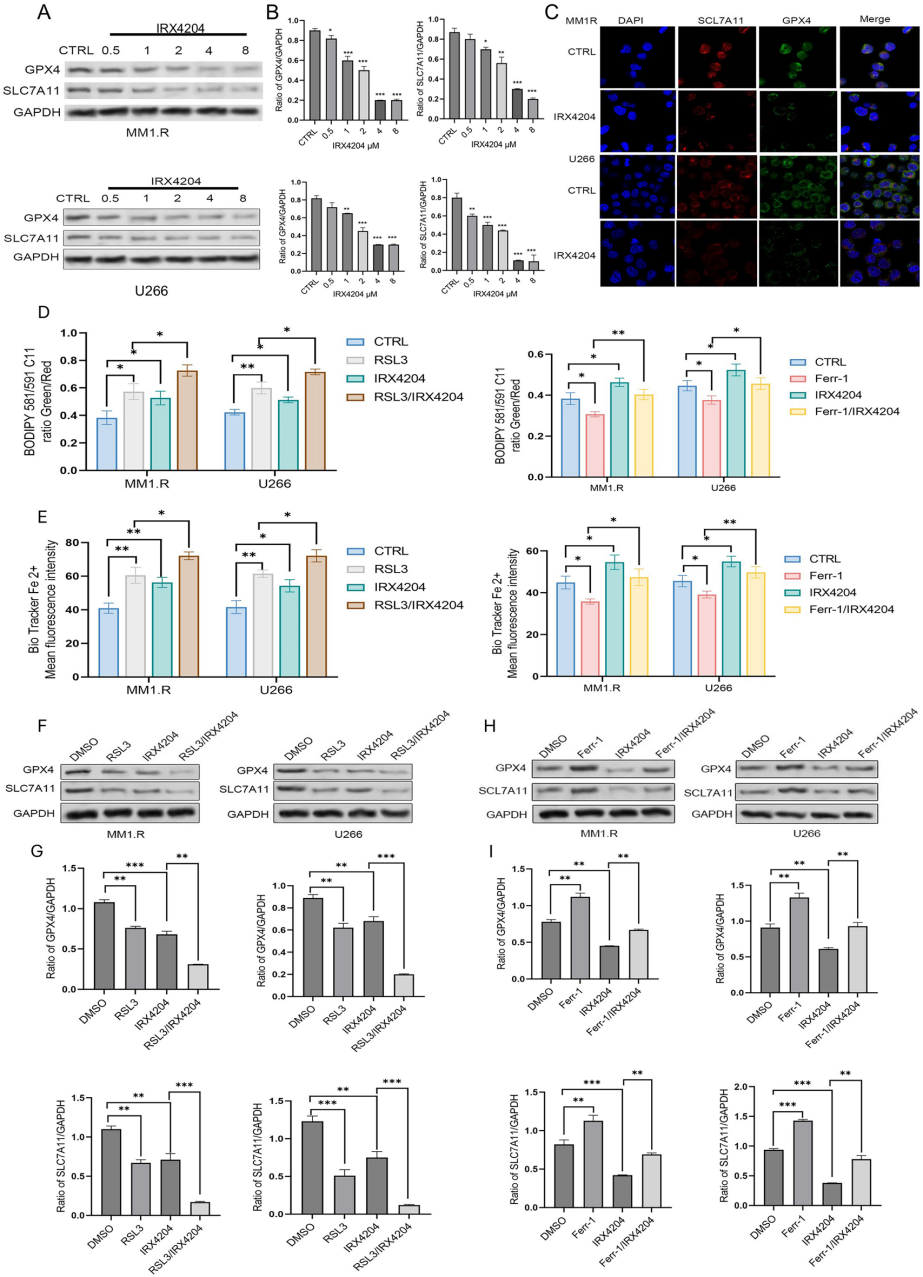
IRX4204 reduces ferroptosis-protective proteins, thereby elevating ferroptotic stress. (**A**) MM1.R and U266 cell lines were treated with the indicated concentrations of IRX4204 for 48 h. Protein lysates were subjected to Western blot analysis. (**B**)The bar graph shows band intensities quantified using ImageJ and normalized to GAPDH control. (**C**) Immunofluorescence staining of GPX4 and SLC7A11 in cells treated with IRX4204 for 48 h. (D) Lipid peroxidation measured by BODIPY C11 probe and flow cytometry analysis. MM1.R cells and U266 cells were treated with DMSO, RSL3 (2 µM), IRX4204 (3 µM) or the combination of RSL3 and IRX4204 (left panel) or DMSO, Ferr-1(2 µM), IRX4204 (3 µM) or the combination of Ferr-1 and IRX4204 (right panel) for 48 h and lipid peroxidation was measured. (**E**) Intracellular ferrous iron levels measured using BioTracker Far-red Labile Fe²⁺ Dye. MM1.R cells and U266 cells were treated with DMSO, RSL3 (2 µM), IRX4204 (3 µM) or the combination of RSL3 and IRX4204 (left panel) or DMSO, Ferr-1(2 µM), IRX4204 (3 µM) or the combination of Ferr-1 and IRX4204 (right panel) for 48 h and intracellular ferrous iron level was measured. (**F**&**G**) MM1.R and U266 cell lines were treated with IRX4204 (3 µM), RSL3 (2 µM), and their combination for 48 h. Protein lysates were subjected to Western blot analysis. The bar graph shows band intensities quantified using ImageJ and normalized to GAPDH control. (**H**&**I**) MM1.R and U266 cell lines were treated with IRX4204 (3 µM), Ferr-1 (2 µM), and their combination for 48 h. Protein lysates were subjected to Western blot analysis. The bar graph shows band intensities quantified using ImageJ and normalized to GAPDH control. Statistical analysis was performed using one-way ANOVA with Dunnett’s post-hoc test for comparisons of all treatment groups versus the control group (Fig. 2C-F). All experiments performed in triplicate; *p < 0.05, **p < 0.01, ***p < 0.001. Full-length, uncropped blots for all western blot images are provided in Supplementary Figure S3. All lanes were from the same gel and in the order shown, no non-adjacent lanes were juxtaposed.

Three key features define ferroptosis: the loss or reduction of phospholipid hydroperoxidase GPX4, oxidation of phospholipids, and the presence of redox-active iron^17^. To understand the molecular mechanisms behind IRX4204-induced ferroptosis, we analyzed ferroptosis-related proteins and cellular stress markers in myeloma cells. Western blot analysis showed that IRX4204 treatment caused a dose-dependent decrease in the levels of the cystine-glutamate antiporter SLC7A11 and the lipid peroxidase GPX4 in both MM1.R and U266 cells (Fig. 2A-B), two important parts of the cell’s antioxidant defense system. Immunofluorescence staining confirmed that IRX4204 significantly lowered the cellular levels of GPX4 and SLC7A11 (Fig. 2C).

Consistent with impaired antioxidant defenses and like RSL3, IRX4204 treatment increased both lipid peroxidation (measured by BODIPY C11 lipid probe, Fig. 2D) and intracellular labile ferrous iron levels (measured by BioTracker Far-red Labile Fe²⁺ Dye, Fig. 2E), which are key signs of ferroptotic stress. Ferr-1 inhibited lipid peroxidation and reduced intracellular labile ferrous iron level (Fig. 2D-E). The combination of IRX4204 and RSL3 caused the greatest reduction in GPX4 and SLC7A11 levels, while co-administration of Ferr-1 largely reversed the IRX4204-induced decrease of these proteins (Fig. 2F-I). This confirms that these molecular changes are functionally linked to ferroptotic cell death rather than non-specific toxicity.

To further characterize the mode of cell death induced by IRX4204, we assessed apoptotic markers in MM cells. Consistent with previous reports showing that IRX4204 can induce apoptosis in other tumor contexts^15^, we observed a measurable but moderate increase in Annexin-positive apoptotic cells following IRX4204 treatment in MM1.R and U266 cells (Supplementary Fig. 2 A). Western blot analysis demonstrated modest induction of cleaved caspase-3 upon IRX4204 treatment (Supplementary Fig. 2B). IRX4204 did not significantly affect cell cycle distribution in either MM1.R or U266 cells (Supplementary Fig. 2 C), indicating that its cytotoxic effect is not primarily mediated by cell-cycle arrest.

Together, these results suggest that IRX4204 engages apoptosis-related pathways in MM cells, while ferroptosis represents the predominant and functionally critical mechanism underlying IRX4204-mediated cytotoxicity under the experimental conditions used in this study.

### Bioinformatic analyses identify HMOX1 as a ferroptosis-related gene with potential RXR regulation

**Fig. 3:**
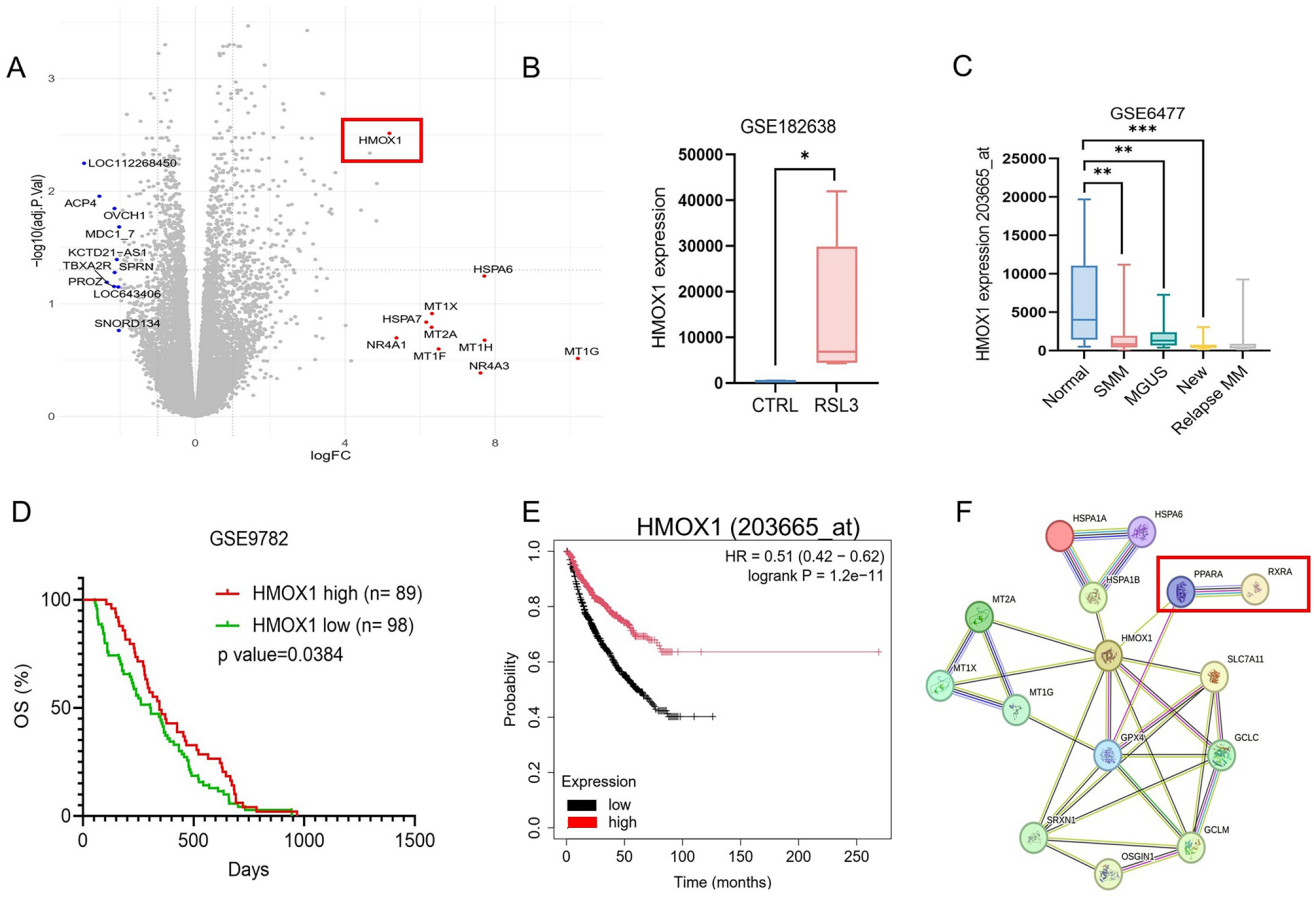
HMOX1 is a ferroptosis-associated gene linked to RXR signaling and clinical outcomes. (**A**) Volcano plot shows HMOX1 among the top upregulated genes in RSL3- treated MM cells. (**B**) HMOX1 expression was elevated in RSL3 treated MM cells compared to control-treated MM cells in GSE182638 dataset. (**C**) HMOX1 decreases across disease stages in GSE6477 dataset: normal donors (ND, *n* = 15), monoclonal gammopathy of undetermined significance (MGUS, *n* = 22), smoldering MM (SMM, *n* = 24), newly diagnosed MM *n* = 73, and Relapse MM *n* = 28. (**D**) Kaplan-Meier survival analysis of MM patients from the APEX clinical trial dataset (GSE9782). **(E**) Patients were stratified into HMOX1-high expression and HMOX1-low groups based on the median HMOX1 expression level. High HMOX1 expression was associated with significantly improved overall survival (OS). (**F**) STRING protein- protein interaction network of ferroptosis-related genes, including HMOX1, GPX4, SLC7A11, PPARA, and RXRA. The network was generated using STRING v 12.0 with a minimum interaction confidence score of 0.7.

To explore the potential molecular mechanisms underlying IRX4204-mediated ferroptosis, we analyzed the GSE182638 dataset, which profiles gene expression changes in myeloma cells treated with the ferroptosis inducer RSL3. Differential expression analysis revealed HMOX1 as one of the most significantly upregulated genes following RSL3 treatment (Fig. 3A), suggesting a potential role for HMOX1 in ferroptosis responses. HMOX1 expression was notably higher in RSL3-treated myeloma cell lines compared to untreated controls (Fig. 3B).

To determine whether HMOX1expression is associated with disease progression, we next analyzed the dataset GSE6477, which included plasma cell transcriptomes from normal donors, MGUS, smoldering MM, newly diagnosed MM, and relapsed MM. HMOX1 levels were significantly higher in normal plasma cells and MGUS but markedly reduced in newly diagnosed MM, with further suppression in relapsed MM (Fig. 3C), indicating progressive HMOX1 suppression during myeloma progression. We further evaluated the clinical relevance of HMOX1 using the GSE9782 (APEX phase III trial) dataset, which includes survival data from relapsed MM patients. Kaplan-Meier analysis demonstrated that patients with high HMOX1 expression had significantly improved overall survival compared with those with low HMOX1 expression (Fig. 3D), supporting a protective role for HMOX1 in MM. To further validate the clinical relevance of HMOX1 expression in MM, we performed an independent survival analysis using the KMplot database (https://kmplot.com/analysis/). Patients were stratified into high and low HMOX1 expression groups based on the optimal cutoff. Consistent with our findings from the APEX cohort, elevated HMOX1 expression was significantly associated with improved overall survival (Fig. 3E). These results further support the protective role of HMOX1 in MM and underscore its potential clinical relevance as a prognostic biomarker.

Protein-protein interaction network analysis using STRING showed that HMOX1 was part of a ferroptosis-related antioxidant regulatory network with predicted interactions with PPARα/RXRα, along with key ferroptosis regulators such as GPX4, SLC7A11, and glutathione biosynthetic enzymes (Fig. 3F). The presence of RXR within the HMOX1 network hierarchy indicated a possible transcriptional regulatory relationship.

Together, these analyses identify HMOX1 as a ferroptosis-related gene that is suppressed during MM progression, associated with better patient outcomes, and potentially regulated by the PPARα/RXRα transcriptional complex.

#### HMOX1 is required for IRX4204-induced ferroptosis in multiple myeloma

**Fig. 4:**
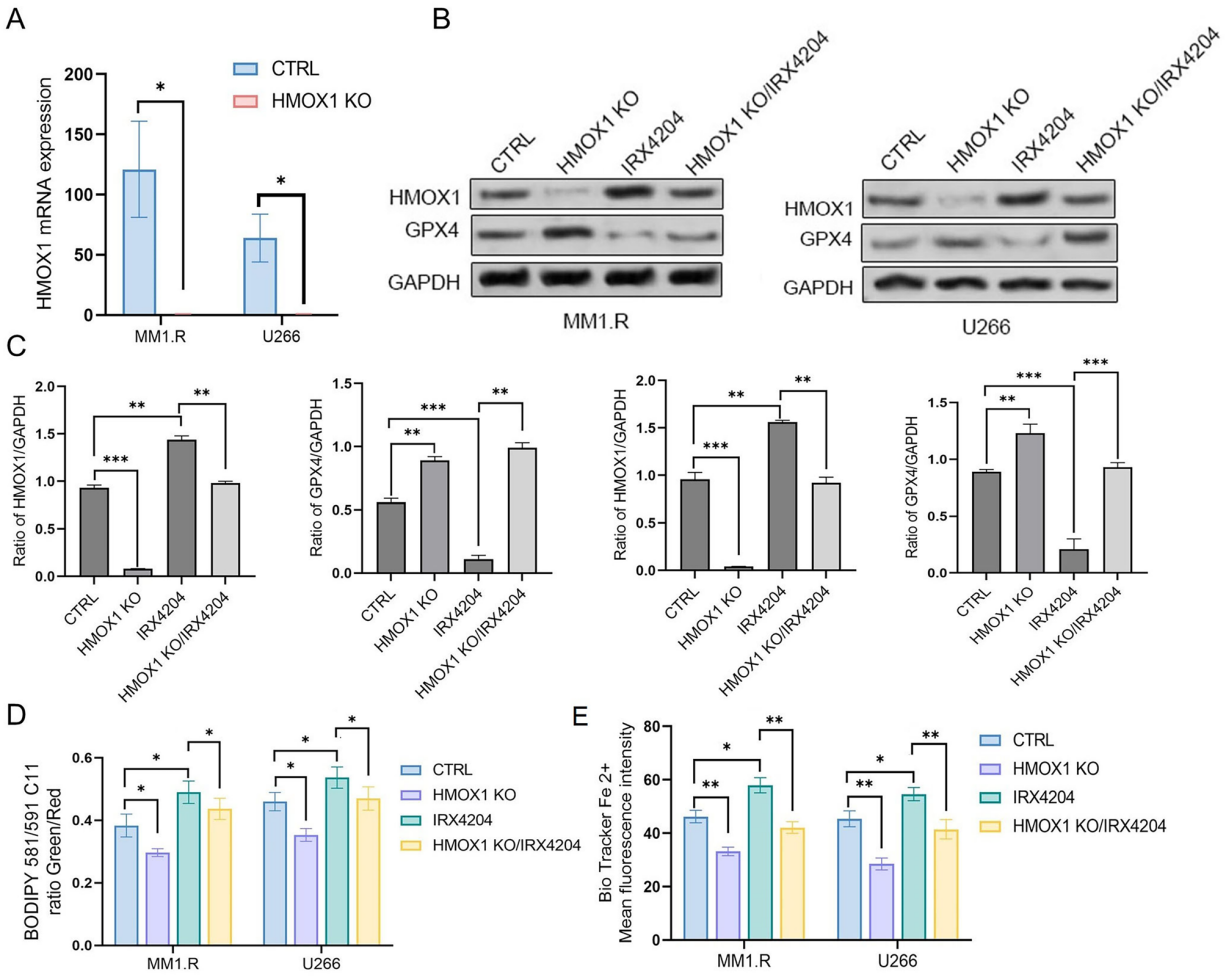
HMOX1 is required for IRX4204-induced ferroptosis (**A**) HMOX1 knockout (KO) validation by qPCR. MM1.R and U266 cells were transduced with HMOX1-specific gRNA CRISPR knockout plasmid and HMOX1 mRNA level was measured by qPCR. (**B**)Western blot analysis of HMOX1 and GPX4 expression in control and HMOX1 knockout MM1.R and U266 cells treated with IRX4204 (3 µM) for 48 h. (**C**) The bar graph shows band intensities quantified using ImageJ and normalized to GAPDH control. (**D**) Lipid peroxidation analysis by BODIPY C11 probe and flow cytometry in control and HMOX1 knockout cells following IRX4204 treatment. Control- and HMOX1 knockout- MM1.R and U266 cells were treated with IRX4204 (3µM) for 48 h and lipid peroxidation was measured. (**E**) Intracellular ferrous iron measurement using BioTracker Far-red Labile Fe²⁺ Dye in control and HMOX1 knockout cells. Control- and HMOX1 knockout- MM1.R and U266 cells were treated with IRX4204 (3µM) for 48 h and intracellular ferrous iron level was measured. Data represent mean ± SD; *p < 0.05, **p < 0.01, ***p < 0.001. Full-length, uncropped blots for all western blot images are provided in Supplementary Figure S5. All lanes were from the same gel and in the order shown; no non-adjacent lanes were juxtaposed.

To determine whether HMOX1 is crucial for IRX4204-induced ferroptosis, we knocked out HMOX1 expression in MM1.R and U266 cell lines using CRISPR/Cas9. HMOX1 deletion was verified at both the mRNA and protein levels (Fig. 4A-C). In wild-type cells, IRX4204 treatment suppressed GPX4 expression. However, knocking out HMOX1 prevented this IRX4204-induced reduction of GPX4, indicating that HMOX1 acts upstream of GPX4 regulation in the ferroptosis pathway (Fig. 4B-C). Functionally, IRX4204 treatment significantly increased lipid peroxidation (Fig. 4D) and intracellular Fe2 + accumulation (Fig. 4E) in wild-type cells, while both effects were markedly reduced in HMOX1-knockout cells, demonstrating that HMOX1 is necessary for IRX4204-mediated ferroptosis in MM.

### IRX4204 transcriptionally activates HMOX1 through PPAR/RXR binding

**Fig. 5:**
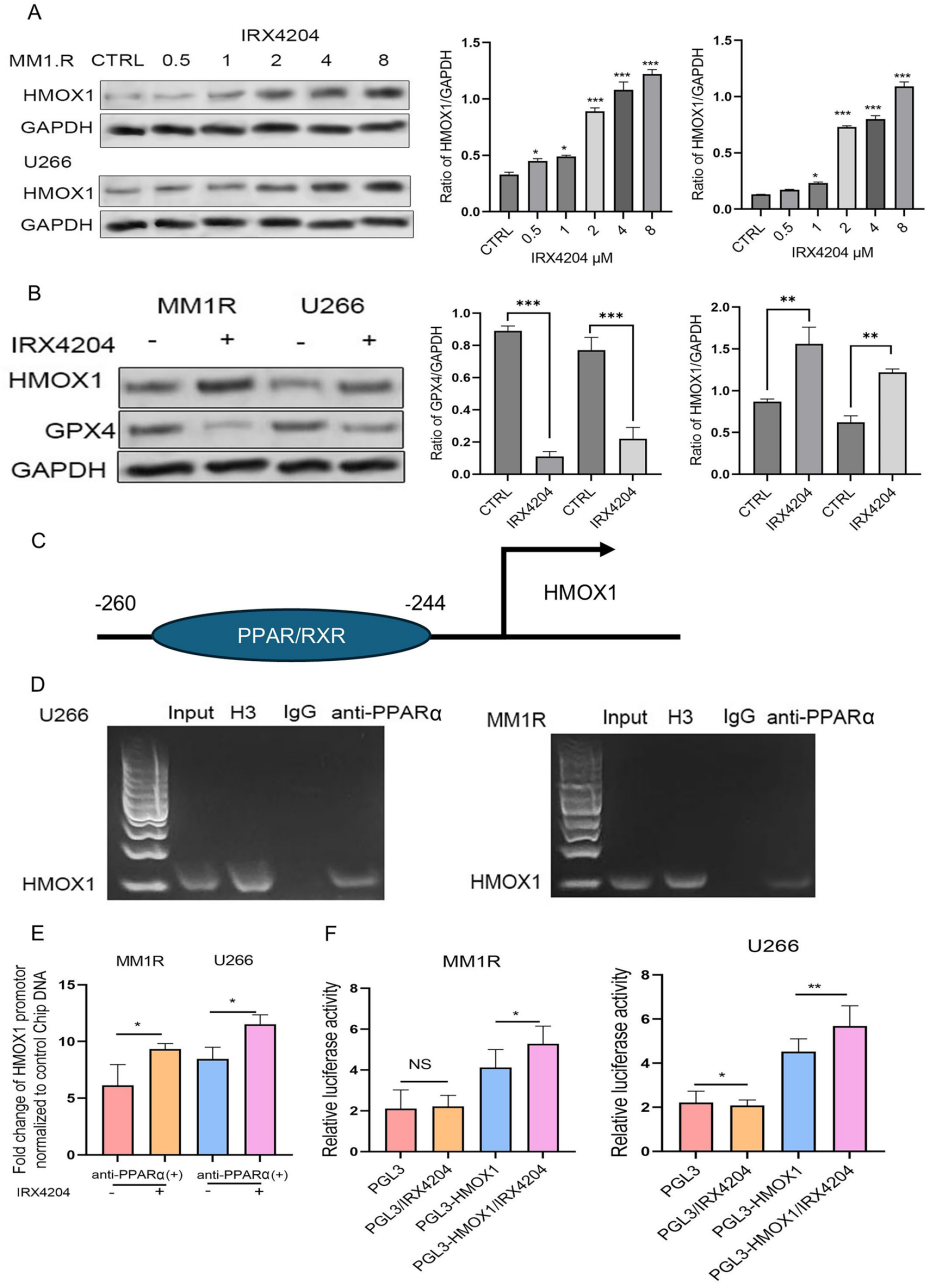
IRX4204 transcriptionally activates HMOX1 through PPAR/RXR binding. (**A**) MM1.R and U266 cells were treated with various concentrations of IRX4204 for 48 h. The protein lysates were subjected to Western blot analysis with the indicated antibodies. The bar graph shows band intensities quantified using ImageJ and normalized to GAPDH control. (**B**) MM1.R and U266 cells were treated with IRX4204 4µM for 48 h. The protein lysates were subjected to Western blot analysis with the indicated antibodies. The bar graph shows band intensities quantified using ImageJ and normalized to GAPDH control. (**C**) JASPAR database prediction of PPAR/RXR binding sites in the HMOX1 promoter region. (**D**) ChIP-PCR gel showing PPARα binding to HMOX1 promoter compared to IgG control in MM1.R and U266 cell lines. (**E**) Quantitative ChIP analysis demonstrating IRX4204-enhanced PPARα/RXRα occupancy of HMOX1 promoter. MM1.R and U266 cell lines were treated with DMSO or IRX4202 4µM for 48 h and PPARα binding to HMOX1 promoter was measured by ChIP-PCR. Statistical analysis was performed using one-way ANOVA followed by Tukey’s multiple comparisons test. (**F**) HMOX1 promoter-driven luciferase reporter assay. Cells were transfected with either PGL3-basic (empty vector) or PGL3-HMOX1 promoter and treated with IRX4204. PGL3-basic with IRX4204 served as a negative control to exclude nonspecific effects of IRX4204 on basal luciferase activity. The predefined primary comparison was PGL3-HMOX1 vs. PGL3-HMOX1 with IRX4204, which was analyzed using an unpaired two-tailed Student’s t -test. Data represent mean ± SD from three independent experiments: *p < 0.05, **p < 0.01, ***p < 0.001. Full-length, uncropped blots for all western blot images are provided in Supplementary Figure S4. All lanes were from the same gel and in the order shown; no non-adjacent lanes were juxtaposed.

IRX4204 treatment increased HMOX1 expression in a dose-dependent manner and concurrently reduced GPX4 levels in MM1.R and U266 cell lines (Fig. 5A–B). Bioinformatic analysis using the JASPAR database indicated that PPARα/RXRα heterodimer could potentially bind to the HMOX1 promoter region (Fig. 5C). Chromatin immunoprecipitation (ChIP) using anti-PPARα confirmed direct binding of the PPARα/RXRα complex to the HMOX1 promoter in both MM1.R and U266 cells, with negligible enrichment in IgG controls (Fig. 5D and E). To determine whether this interaction activates HMOX1 transcription, we cloned the HMOX1 promoter into a luciferase reporter construct. IRX4204 treatment significantly increased HMOX1 promoter–driven luciferase activity compared to control, consistent with transcriptional activation of HMOX1(Fig. 5F). IRX4204 directly activates HMOX1 transcription through PPARα/RXRα biding to its promoter, thereby connecting RXR signaling to ferroptosis regulation in MM.

### IRX4204 enhances lenalidomide antitumor efficacy in vivo through ferroptosis activation

**Fig. 6:**
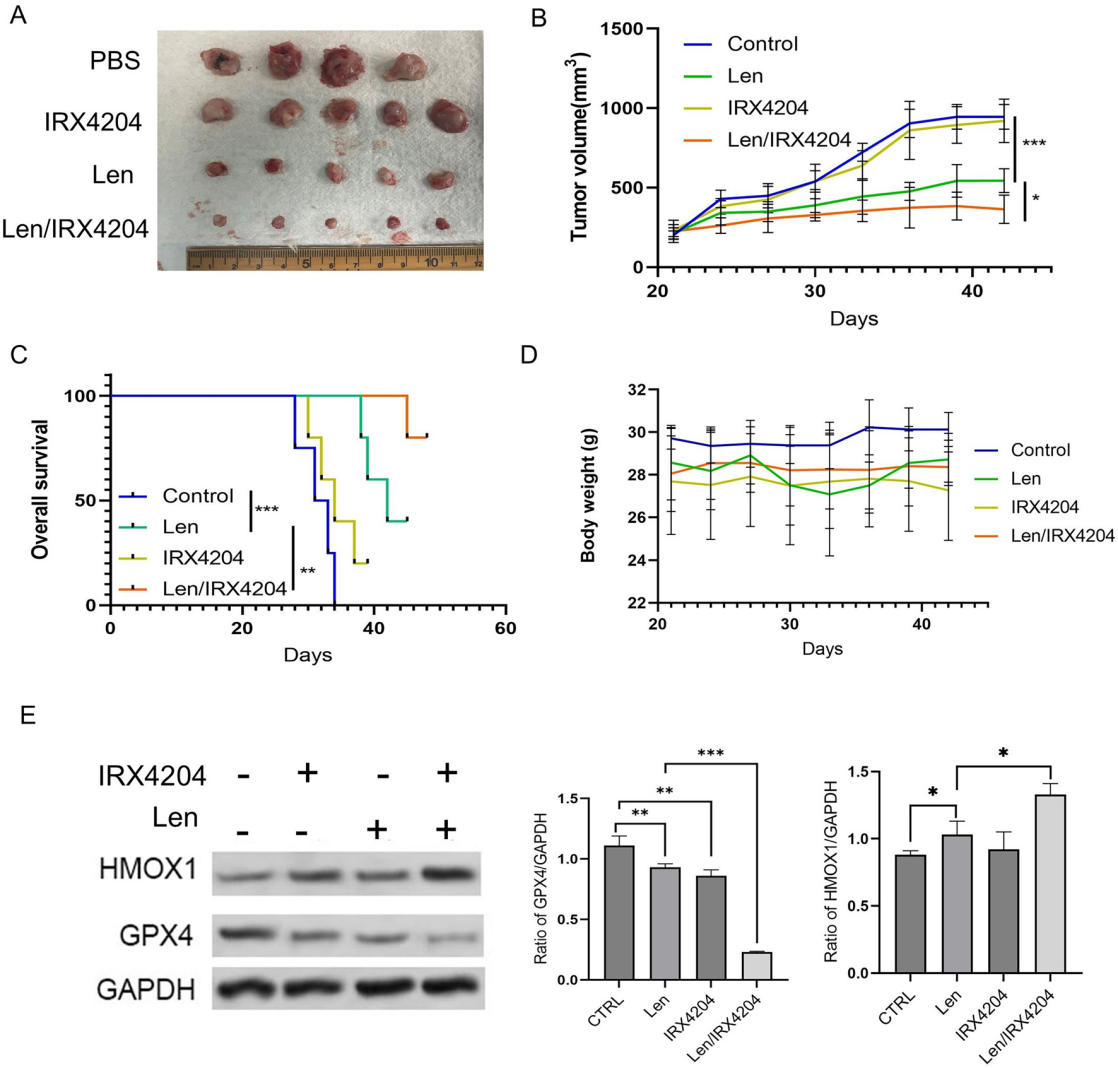
IRX4204 enhances lenalidomide-mediated anti-myeloma efficacy in vivo by promoting ferroptosis. (**A**) Representative images of SCID mice with subcutaneous multiple myeloma tumor. (**B**) IRX4204 enhanced lenalidomide-induced attenuations of tumor growth in severe combined immunodeficient mice. Statistical analysis was performed using two-way ANOVA with Sidak’s multiple comparisons test. (**C**) OS was evaluated using the Kaplan-Meier method and the log-rank test from the first day of tumor cell injection until death or the occurrence of an event. Survival differences were analyzed using the log-rank (Mantel-Cox) test. (**D**) Body weight was measured every 3 days and presented as means ± SD. (**E**) Tumors treated as above were analyzed by immunoblotting with the indicated antibodies. The bar graph shows band intensities quantified using ImageJ and normalized to GAPDH control. Full-length, uncropped blots for all western blot images are provided in Supplementary Figure S5. All lanes were from the same gel and in the order shown; no non-adjacent lanes were juxtaposed.

Having established that IRX4204 induces ferroptosis via the RXR-HMOX1-GPX4 pathway in vitro, we assessed whether this mechanism translates into therapeutic effectiveness in vivo. We developed subcutaneous MM1.R xenografts in NSG mice and treated the animals with IRX4204 (10 mg/kg IP, five times weekly), lenalidomide (10 mg/kg/day), or the combination. The combination therapy resulted in significantly smaller tumors than either single agent or control groups (Fig. 6A-B) and extended survival (Fig. 6C). No adverse toxicity was observed, as shown by stable body weight throughout treatment (Fig. 6D). To verify that the therapeutic effect involved ferroptosis activation, we analyzed tumor tissues for ferroptosis markers. Tumors treated with the combination exhibited increased HMOX1 and decreased GPX4 levels compared to lenalidomide alone or PBS controls (Fig. 6E), aligning with our in vitro findings. These results demonstrate that IRX4204 boosts lenalidomide efficacy through ferroptosis activation in vivo.

## Discussion

Multiple myeloma (MM) remains an incurable cancer for most patients, mainly because of therapeutic resistance and the inability to eliminate metabolically adaptable plasma cells^18,19^. In this study, we uncover a previously unknown role of retinoid X receptor (RXR) signaling in controlling ferroptosis and show that the selective RXR agonist IRX4204 activates a transcriptional program that primes MM cells for ferroptosis-dependent death. IRX4204 downregulates GPX4 and SLC7A11, increases lipid peroxidation and labile Fe²⁺ levels, and works synergistically with RSL3 and lenalidomide in vitro. Mechanistically, we demonstrate that IRX4204 promotes PPARα/RXRα binding to the HMOX1 promoter, leading to increased HMOX1 transcription and subsequent GPX4 depletion. CRISPR-mediated deletion of HMOX1 prevents lipid peroxidation, iron buildup, and GPX4 suppression, confirming HMOX1 as a key component of this pathway. In vivo, IRX4204 enhances lenalidomide’s activity and induces molecular changes consistent with ferroptosis, supporting its potential for clinical translation.

Ferroptosis has become an important form of regulated cell death driven by iron-dependent lipid peroxidation^20,21^. Hematologic cancers, including MM, show increased sensitivity to ferroptosis because of their active mitochondrial metabolism, iron flux, and reliance on glutathione-based antioxidant systems^22^. Earlier studies have shown that suppressing GPX4 or system Xc⁻ can induce ferroptosis and produce antitumor effects, although most methods depend on direct enzymatic inhibition, which often raises concerns about toxicity^23,24^. Growing evidence emphasizes the dual, context-dependent roles of HMOX1: while traditionally cytoprotective, HMOX1 can promote ferroptosis when iron release exceeds sequestration capacity^25,26^. This pro-ferroptotic role has been observed in some solid tumors but is still poorly understood in hematologic malignancies. Our findings expand this emerging framework by demonstrating that transcriptional activation of HMOX1 via nuclear receptor signaling is sufficient to induce ferroptosis in MM.

Parallel to these developments, RXR biology in cancer has gained renewed attention^27^. RXR functions as a heterodimeric partner for nuclear receptors such as PPARs, LXRs, and RARs, and it participates in metabolic regulation, differentiation, and immune signaling^28,29^. Third-generation RXR agonists like IRX4204 demonstrate significantly improved receptor specificity, reducing off-target RAR activation and allowing more precise transcriptional control^30^. Prior research has mainly focused on the immunomodulatory and metabolic effects of RXR agonists in hematologic malignancies^31,32^. However, nuclear receptors are increasingly recognized as regulators of redox metabolism and iron homeostasis, indicating their role in ferroptosis pathways. Our study offers direct evidence for this idea by identifying RXR as a transcriptional regulator of ferroptotic susceptibility through its control of the HMOX1–GPX4 axis.

Several unexpected observations emerged from this study. Most notably, HMOX1 proved essential for IRX4204-induced ferroptosis, despite its traditional role in protecting cells. This highlights the context-dependent nature of HMOX1 biology and demonstrates how the unique metabolic traits of MM, such as high iron turnover and oxidative stress, can lead HMOX1 to promote ferroptosis^12,33^. Additionally, the strong synergy between IRX4204 and lenalidomide suggests that oxidative stress from immunomodulatory therapy may be more impactful than previously thought when antioxidant defenses are reduced. These findings reveal hidden vulnerabilities that can be uncovered through transcriptional reprogramming.

A deeper mechanistic understanding of how HMOX1 regulates GPX4 expression in ferroptosis strengthens our biological framework. Although HMOX1 is not known to repress GPX4 transcription directly, accumulating evidence indicates that HMOX1 indirectly suppresses GPX4 by inducing iron-mediated oxidative stress, which drives ferroptosis^34^. Mechanistically, HMOX1 enzymatically breaks down heme into biliverdin, carbon monoxide, and free ferrous iron^35,36^. When HMOX1 is highly activated, the rapid release of Fe²⁺ exceeds the capacity of ferritin and ferroportin to sequester it, leading to an increase in the labile iron pool^37,38^. The elevated Fe²⁺ catalyzes Fenton chemistry, producing hydroxyl radicals and intense lipid ROS^39,40^. This oxidative environment destabilizes GPX4 by oxidizing key selenocysteine and cysteine residues, promoting its degradation and impairing its enzymatic function^41^. Additionally, severe ROS stress can downregulate GPX4 mRNA via stress-responsive pathways like ATF3, NF-κB, and microRNA-mediated repression^42^. Collectively, these events cause the collapse of the GPX4–GSH antioxidant system. Notably, this iron-driven suppression of GPX4 has been observed in models of osteosarcoma, liver cancer, and small-cell lung cancer, indicating a conserved mechanism across tumor types^43–45^. Consistent with these findings, our genetic data showing that HMOX1 deletion completely prevents GPX4 reduction confirms HMOX1 as an essential upstream trigger of GPX4 loss in MM cells exposed to IRX4204.

Our findings enhance current ferroptosis models by identifying RXR signaling as a key transcriptional factor influencing ferroptosis sensitivity. While traditional models focus on metabolic regulators like glutathione levels, lipid makeup, and iron loading, this study shows that transcriptional networks, especially nuclear receptor pathways, determine the baseline vulnerability to ferroptosis by regulating antioxidant responses, iron handling, and lipid peroxidation^46,47^. We propose a model in which RXR activation induces a transcriptionally active state characterized by increased HMOX1 and decreased GPX4, thereby lowering the threshold for ferroptosis under oxidative stress. This mechanistic framework supports combining IRX4204 with lenalidomide and potentially other redox-based or immunotherapies.

While IRX4204 demonstrated robust ferroptosis-inducing effects in vitro, its in vivo anti-tumor effects on tumor volume were modest though accompanied by significant survival benefit in combination with lenalidomide (Fig. 6). This apparent discrepancy reflects several important factors. First, ferroptosis-mediated tumor suppression operates through mechanisms distinct from apoptotic agents, primarily impairing tumor metabolic fitness and disrupting redox homeostasis rather than inducing rapid cytotoxic cell death^48,49^. This metabolic disruption manifests as limited tumor progression and enhanced survival, effects better captured by long-term survival endpoints than short-term volumetric measurements^49^. Second, pharmacokinetic factors including drug bioavailability, tumor penetration, and in vivo metabolism may differ substantially from in vitro conditions^50,51^. Our study evaluated a single dose and schedule; systematic dose-optimization studies, including alternative dosing regimens and formulations, may yield more robust volumetric responses as demonstrated with other ferroptosis modulators^52,53^. The combination therapy with lenalidomide demonstrated superior efficacy, positioning IRX4204 as a sensitizing agent that primes cells to ferroptotic stress while enhancing the effects of standard therapies. These findings establish mechanistic feasibility and identify clear paths for therapeutic optimization through dose-finding studies, alternative schedules, and immunocompetent models.

This study has several limitations. The in vivo work was conducted in immunodeficient mice, which prevents the assessment of how IRX4204-induced ferroptosis might interact with the immune microenvironment. Additionally, although HMOX1 and GPX4 emerged as key regulators, the broader transcriptional changes induced by RXR activation remain undefined. Further research using immunocompetent models and patient-derived samples will be needed to fully evaluate the therapeutic potential.

## Methods

### Animal ethics

All animal experiments in this study were carried out in accordance with procedures approved by the Duke University Institutional Animal Care and Use Committee under protocol A073-23-03, and were performed in accordance with institutional guidelines. The study is reported in accordance with ARRIVE guidelines.

### Cell lines

The human myeloma cell lines MM1.R, U266 and RPMI8226 were sourced from the American Type Culture Collection (Manassas, VA). Short tandem repeat analysis was performed to verify their genetic identity. Cells were maintained in RPMI-1640 as the base medium, containing 10% fetal bovine serum, 2 mM GlutaMAX, and 1% penicillin–streptomycin, and were cultured under standard conditions.

### Antibodies and reagents

This study employed several commercially available antibodies and small-molecule reagents. Antibodies included those specific for SLC7A11 (PA5-116134, Invitrogen), GPX4 (67763-1-Ig, Proteintech), GAPDH (2118, Cell Signaling Technology), and HMOX1 (E7U4W, Cell Signaling Technology). The ferroptosis-related compounds RSL3 (HY-100218 A) and Ferrostatin-1 (HY-15763) were purchased from MedChemExpress. IRX4204 was supplied by Io Therapeutics, Inc. through a material transfer agreement and was prepared in DMSO for experimental use.

### Cell viability

Cell viability was assessed using an MTT-based approach. In brief, 5 × 10⁴ cells were placed into each well of a 96-well plate (three replicates per condition) in 100 µL of culture medium containing the indicated doses of IRX4204 or RSL3. MM cells were treated with RSL3/IRX4204 or Ferr-1/IRX4204 simultaneously (i.e., drugs added at the same time with no pretreatment period) unless otherwise stated. The plates were incubated at 37 °C with 5% CO₂ for the required time. After incubation, 20 µL of the MTS/PMS mixture (equivalent to 5 mg/mL MTT; Promega G5421) was added, and cells were allowed to react for 3–4 h. Absorbance at 490 nm was then measured on a VERSAmax microplate reader (Molecular Devices).

### Western blotting

Western blot was carried out following previously published procedures^7^ with minor modifications. Cells were lysed using a modified 4× Laemmli buffer supplemented with protease inhibitors, and protein concentrations were quantified with the DC protein assay (BioRad). Equal protein amounts (20 µg) were resolved by SDS-PAGE, transferred onto PVDF membranes, and incubated overnight at 4 °C with primary antibodies. Fluorescent secondary antibodies (catalog no: 926–68072, 926–68071; IRDye, LiCor) were used for detection, and signals were visualized on an Odyssey CLX imaging system. Band intensities were quantified using ImageJ software.

### Immunofluorescence confocal microscopy

For confocal imaging, cells were cytospun onto slides and fixed in 4% formaldehyde for 15 min. Following fixation, samples were blocked with medium containing 10% FBS and incubated overnight at 4 °C with primary antibodies against SLC7A11 or GPX4. After triple PBS washes, slides were incubated for 1 h with Alexa Fluor 594 anti-rabbit secondary antibody (Thermo Fisher, R37117). Nuclei were stained with DAPI (Cell Signaling 4083) and coverslipped with antifade medium (Vector Laboratories). Images were obtained using a Leica SP5 confocal system equipped with a 63×/1.1 oil objective at 512 × 512 pixel resolution.

### Ferrous iron determination

To quantify intracellular ferrous iron, 2 × 10⁵ cells were washed twice in PBS and then stained with 5 µM BioTracker Far-red Fe²⁺ dye (Millipore SCT037) for 90 min at 37 °C in the dark. Cells were rinsed, suspended in PBS, and fluorescence signals were recorded using a Cytation 5 imaging reader (Agilent). Intensities were processed using ImageJ.

### Lipid peroxidation assay

Lipid peroxidation was measured using the BODIPY 581/591 C11 probe (Invitrogen). Cells were incubated with 5 µM dye for 30 min at 37 °C, washed, and analyzed on a BD FACSCanto II cytometer. The relative oxidation level was calculated from the ratio of FL1 to FL3 fluorescence.

### Chromatin immunoprecipitation (ChIP) assay

ChIP assays were performed using the Simple Chip kit (91820, Cell Signaling Technology). Cells were cross-linked with 1% formaldehyde, pelleted, and sonicated. Chromatin was precleared with 30 µL of ChIP-Grade protein G magnetic beads (9006, Cell Signaling Technology), and incubated with antibodies against histone H3 rabbit antibody, normal rabbit IgG, and anti-PPARα antibody (ab227074; Abcam). Purified ChIP DNA was analyzed by qPCR using HMOX1 promoter primers: forward CCACCTCCACCTTCCCTTAA and reverse GGGAGATAGGGAATGCAGACA. Enrichment was calculated relative to input DNA and normalized to IgG controls.

### HMOX1 promoter-luciferase reporter assay

Genomic DNA was isolated using the DNeasy purification system (Qiagen). The upstream regulatory region of the **HMOX1** gene was amplified by PCR with gene-specific primers (forward: TGCCAAGAGATTACCTGGGG; reverse: AGGGGAGAAGGGAGATAGGG). The amplified fragments were treated with the restriction enzymes NheI and XbaI, resolved on a 1% agarose gel, and recovered with a gel extraction kit. The purified inserts were ligated into the pGL3 firefly luciferase reporter backbone. MM1.R and U266 cells were plated at 1 × 10⁴ cells per well and transfected with the HMOX1–luciferase construct using Lipofectamine 3000. After a 48-hour incubation, luciferase signals were quantified using the Dual-Luciferase Reporter Assay System (Promega E1910).

### CRIPSR-Cas9 based HMOX1 knock out

HMOX1-deficient MM cells were produced with CRISPR–Cas9 technology. Single-guide RNAs targeting HMOX1 were inserted into the pX330-U6-Chimeric_BB-CBh-hSpCas9 plasmid or the lentiCRISPR v1 backbone (Addgene). Single-guide RNAs (gRNAs) targeting HMOX1 were designed using the CHOPCHOP v3 web tool (https://chopchop.cbu.uib.no/), which integrates multiple scoring algorithms to evaluate on-target efficiency and off-target potential. The sgRNA sequence used in this study (CGCAACCCGACAGGCAAGCGCGG) was ranked among the top candidates by CHOPCHOP and showed no predicted high-risk off-target sites with fewer than three mismatches in coding regions. The top predicted off-target sites are listed in Supplementary Table 1. Following transfection or lentiviral delivery, edited cells were expanded, and the loss of HMOX1 protein expression was verified by immunoblotting^54^.

### Myeloma xenograft mouse model

NSG mice (both male and female, 6–8 weeks old; The Jackson Laboratory) weighing 18–22 g were used to establish subcutaneous myeloma tumors. Mice were housed in a specific pathogen-free barrier facility and fed standard chow. All procedures were approved by the Duke University Institutional Animal Care and use Committee (IACUC protocol A073-23-03) and conducted in accordance with ARRIVE guidelines. A total of 19 mice were used in the study and assigned to four groups. PBS control (*n* = 4), IRX4204 (*n* = 5), lenalidomide (*n* = 5), and combination IRX4204 with lenalidomide (*n* = 5). No animals were excluded from the analysis unless humane endpoints were met. Investigators performing tumor measurements were blinded to group assignments throughout the study.

1 × 10⁷ MM1.R cells suspended in 100 µl of serum-free RPMI were injected subcutaneously into the right flank of each animal. Tumors became detectable approximately three weeks after implantation, at which point mice were randomly assigned to receive lenalidomide (10 mg/kg/day, intraperitoneally), IRX4204 (10 mg/kg, intraperitoneally, five times per week), combination therapy, or vehicle control. Tumor progression was monitored every three days using calipers, and volumes were estimated with the formula: 0.5 ×(length × width²). IRX4204 was obtained from Io Therapeutics and dissolved in DMSO. Body weight, grooming behavior, posture, and overall activity were monitored daily to assess treatment-related toxicity. Mice were euthanized when humane endpoints were met, including excessive tumor size (> 1.5 cm), paralysis, or signs of significant distress. Euthanasia was performed using a gradual-fill CO2 inhalation method from a compressed gas cylinder at a displacement rate of 20–30% of the chamber volume per minute, following AVMA and Duke IACUC guidelines. A secondary physical method (cervical dislocation) was used to confirm death. All euthanasia procedures were carried out using IACUC-approved protocols and equipment. Tumor tissues were harvested for protein analysis. All in vivo procedures conformed to approved IACUC protocols.

### STRING interaction network analysis

To explore the interaction network linking HMOX1 with ferroptosis and redox-related regulatory pathways, a protein-protein interaction (PPI) network analysis was constructed using the STRING database (version 12.0; https://string-db.org). The input gene list was curated based on ferroptosis-related genes from the FerrDb database, published literature, and included key regulators of iron metabolism, lipid peroxidation, and antioxidant defense pathway: HMOX, GPX4, SLC7A11, MT2A, PPAR α, and RXR α. The minimum interaction confidence score was set to 0.7 (high confidence). The resulting network was visualized using STRING and exported for figure generation.

#### Bioinformatic analysis

Gene expression datasets related to ferroptosis induction, myeloma disease progression, and patient survival were retrieved from the Gene Expression Omnibus (GEO) database. The dataset GSE182638 was used to identify transcriptional responses associated with ferroptosis induction in MM cells following treatment with GPX4 inhibitor RSL3. Differentially expressed genes were identified using GEO2R with Benjamini-Hochberg correction. Genes with adjusted *p* < 0.05 and fold change > 1 were considered significant.

Clinical outcomes analysis was performed using the GSE9782 (APEX phase III trial) dataset, which contains transcriptomic profiles and overall survival data from patients with relapsed MM treated with bortezomib or dexamethasone. Patients were dichotomized into high and low HMOX1 expression groups based on median expression values. Kaplan-Meier survival analysis was conducted using the log-rank test.

### Statistical analysis

Data are presented as mean ± standard deviation (SD) from at least three independent experiments. For comparisons involving more than two groups, one-way or two-way ANOVA was used as appropriate, followed by Dunnett’s or Sidak’s multiple comparisons test. For two-group comparisons, an unpaired two-tailed Student’s T-test was applied. Kaplan-Meier survival curves were analyzed by the log-rank test. Statistical analyses were performed using GraphPad Prism v9 (GraphPad, Inc), and significance was defined as *p* < 0.05. Significance levels are indicated as: *p < 0.05; **p < 0.01; ***p < 0.001; ****p < 0.0001.

## Supplementary Information

Below is the link to the electronic supplementary material.

Supplementary Material 1

## Data Availability

The data presented in this study are available on request from the corresponding author.
