# Supplementary material for: IRX4204 sensitizes multiple myeloma to ferroptosis and improves lenalidomide efficacy through the HMOX1-GPX4 axis
Source: Sci Rep. 2026 Mar 17;16:13832. doi: 10.1038/s41598-026-42123-9 (PMC13128853; doi:10.1038/s41598-026-42123-9)
Supplement: Supplementary file 1 — Supplementary Material 1 [file 41598_2026_42123_MOESM1_ESM.docx]

**Title: IRX4204 sensitizes multiple myeloma to ferroptosis and improves lenalidomide efficacy through the HMOX1-GPX4 axis**

**Author information**

Jian Wu¹, Zhibo Yan², Kimberly Burcher¹, Zhannan Han², Mikhail A. Nikiforov²^,^³, Vidyasagar Vuligonda^4^, Martin Sanders^4^, Yubin Kang¹*

¹Division of Hematologic Malignancies and Cellular Therapy, Department of Medicine, Duke University Medical Center, Durham, NC 27710, United States.

²Department of Pathology, Duke University School of Medicine, Durham, NC 27710, United States.

³Department of Biomedical Engineering, Pratt School of Engineering, Duke University, Durham, NC 27708, United States.

^4^Io Therapeutics, Inc., Spring, TX 77387, United States.

**^*^Corresponding author:** Yubin Kang, MD, Duke University Medical Center, DUMC Box 3961, Durham, NC 27710, United States. Tel: (919) 668-2331. Email: yubin.kang@duke.edu

Supplementary Table 1. Top predicted off-target sites.

| Rank | Target sequence | Genomic location | Strand | GC content (%) | MM0 | MM1 | MM2 | MM3 | Efficiency |
| --- | --- | --- | --- | --- | --- | --- | --- | --- | --- |
| 1 | CGCAACCCGACAGGCAAGCGCGG | chr22:35381184 | + | 70 | 0 | 0 | 0 | 0 | 50.32 |
| 2 | CGAGACGGCTTCAAGGTATGTGG | chr22:35383212 | + | 55 | 0 | 0 | 0 | 1 | 58 |
| 3 | TTGCGGACGCTCCATCCGGCCGG | chr22:35381166 | - | 70 | 0 | 0 | 0 | 1 | 52.65 |
| 4 | GCAACCCGACAGGCAAGCGCGGG | chr22:35381185 | + | 70 | 0 | 0 | 0 | 1 | 48.42 |
| 5 | GTAAGGACCCATCGGAGAAGCGG | chr22:35393527 | - | 55 | 0 | 0 | 1 | 0 | 58.74 |


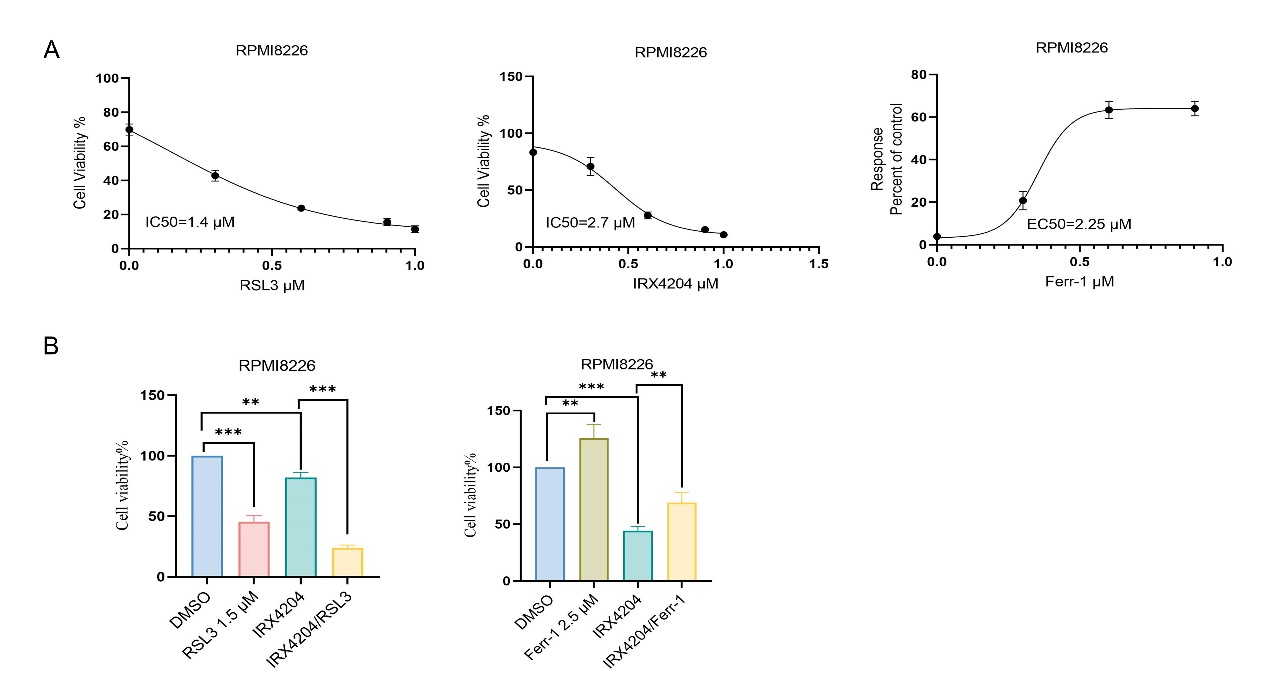


**Supplementary Figure 1. IRX4204 sensitizes multiple myeloma cells to ferroptosis.** (A) Dose–response curves of RPMI8226 MM cells treated with IRX4204, RSL3, or Ferr-1. (B) RPMI8226 MM cells were treated with DMSO, RSL3, IRX4204 (3 μM), or the combination and incubated for 48 h. Cell viability was assessed by the MTT assay. All experiments performed in triplicate; *p<0.05, **p<0.01, ***p<0.001.


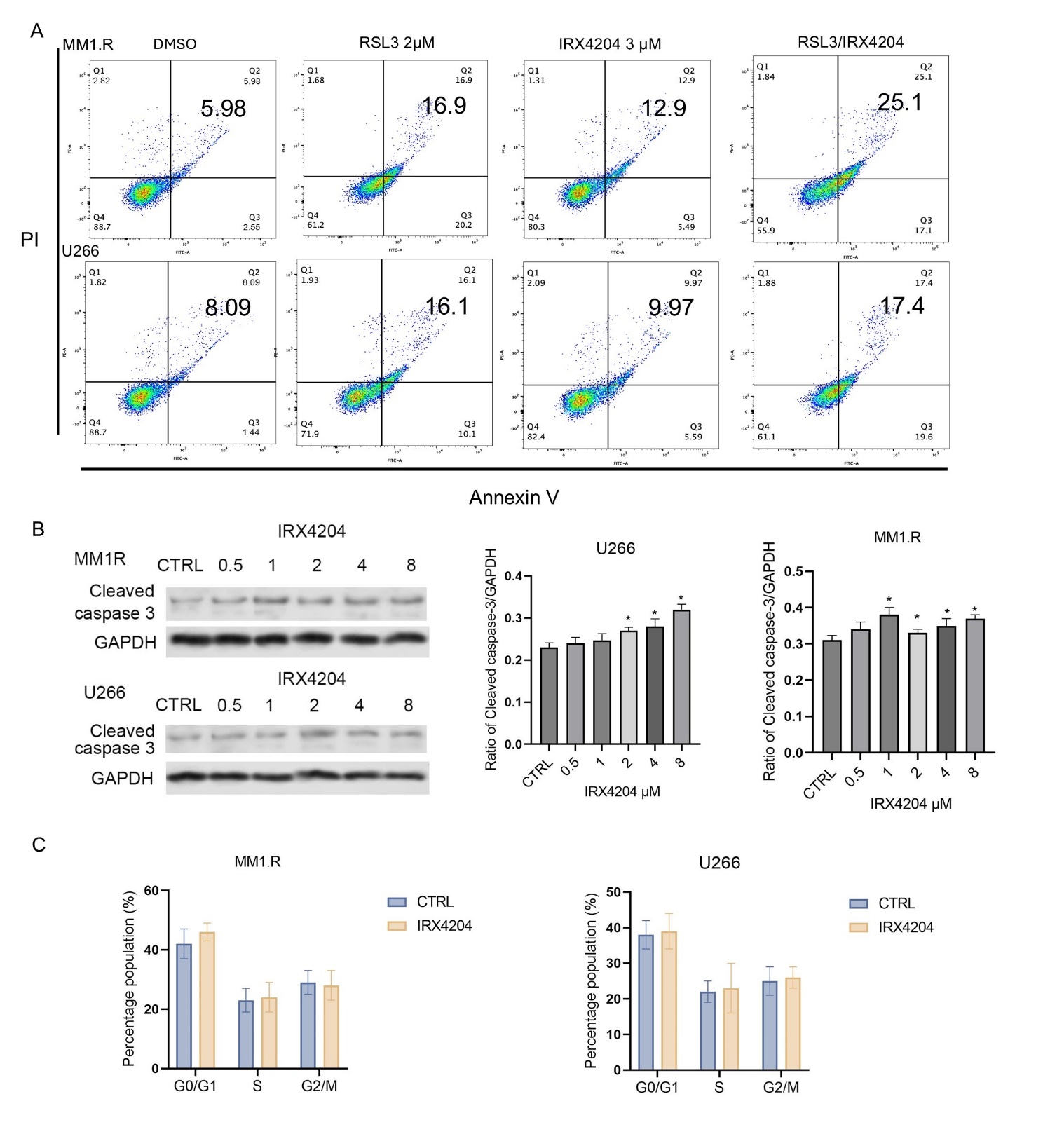


**Supplementary Figure 2. IRX4204 induces apoptosis-associated features in MM cells.** (A) MM1.R and U266 cells were treated with DMSO, RSL3, IRX4204, or the combination for 48h. Apoptosis was assessed by Annexin V/PI staining followed by flow cytometry. (B). MM1.R and U266 cells were treated with increasing concentrations of IRX4204 for 48h. Cell lysates were subjected to western blot analysis for cleaved caspase-3. Data shown are representative of three independent experiments. The bar graph shows band intensities quantified using ImageJ and normalized to GAPDH control. (C). Cell cycle distribution of MM1.R and U266 cells treated with IRX4204 was analyzed by flow cytometry. The percentages of cells in G0/G1, S, and G2/M phases are shown as Mean ± SD from three independent experiments.


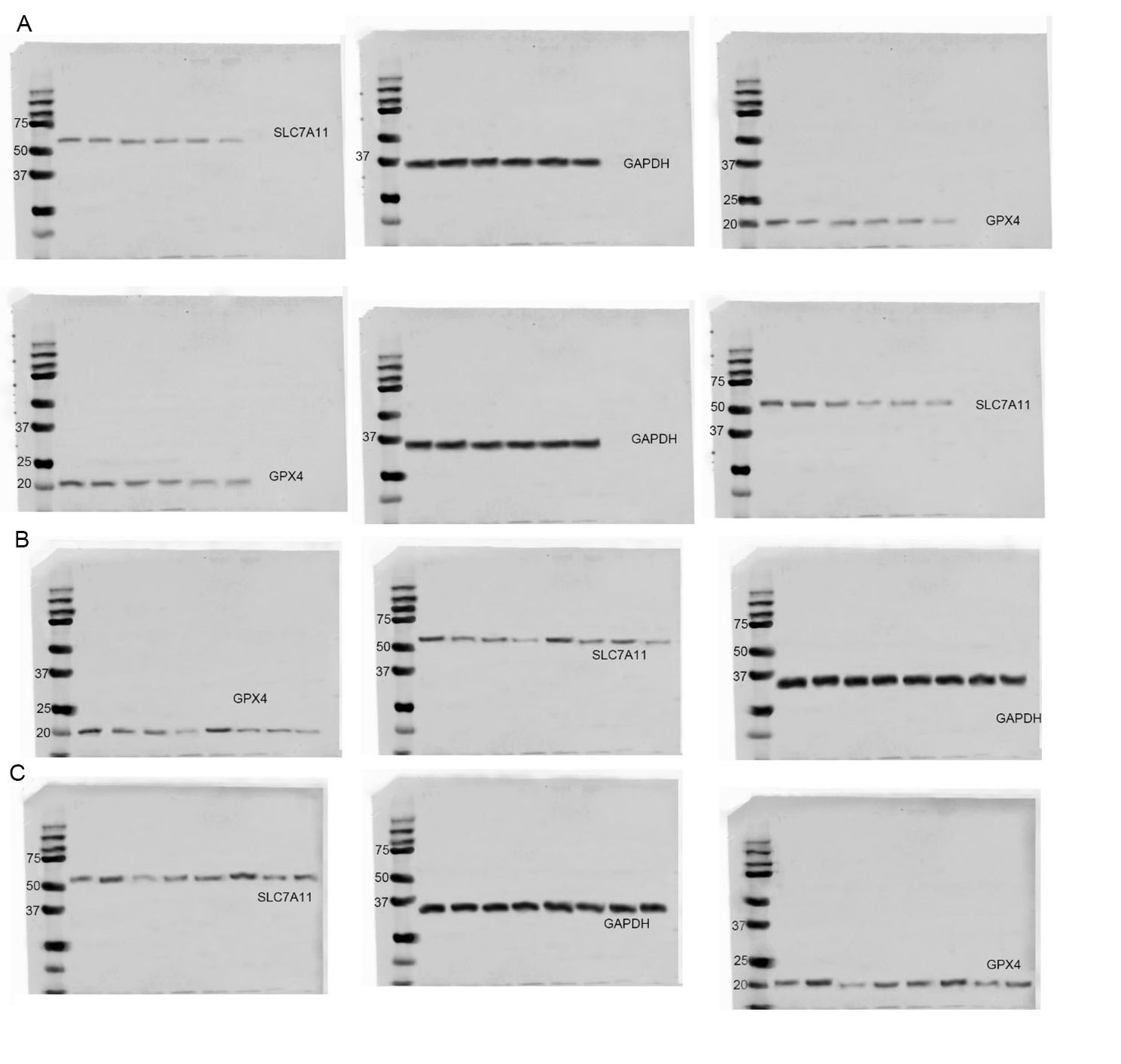


**Supplementary Figure 3. Uncropped western blot images.**

(A-C) Full length, uncropped blots corresponding to Figure 2A, 2C, and 2D, respectively.


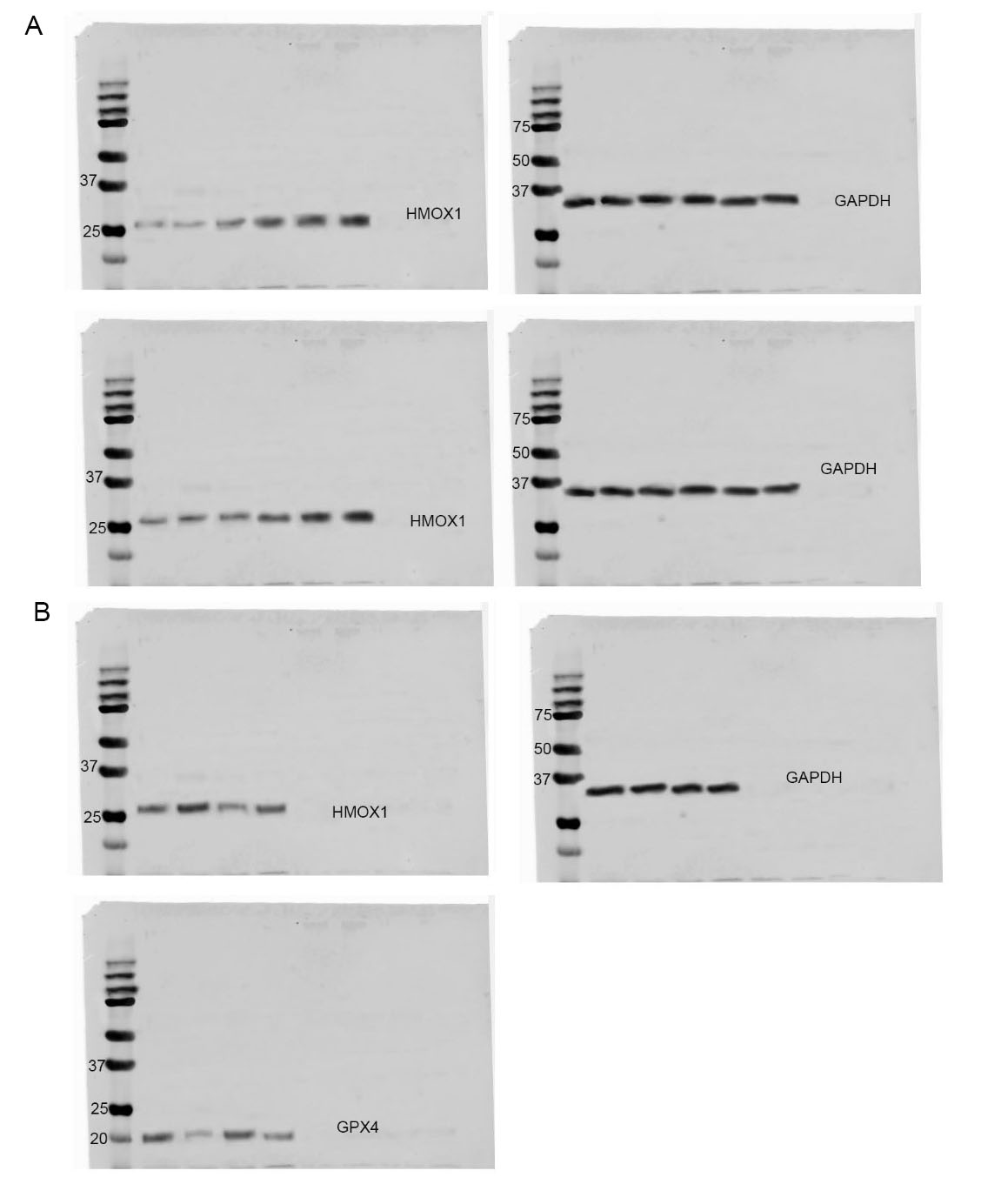


**Supplementary Figure 4. Uncropped western blot images.**

(A-B) Full-length, uncropped blots corresponding to Figure 5A and 5B, respectively.


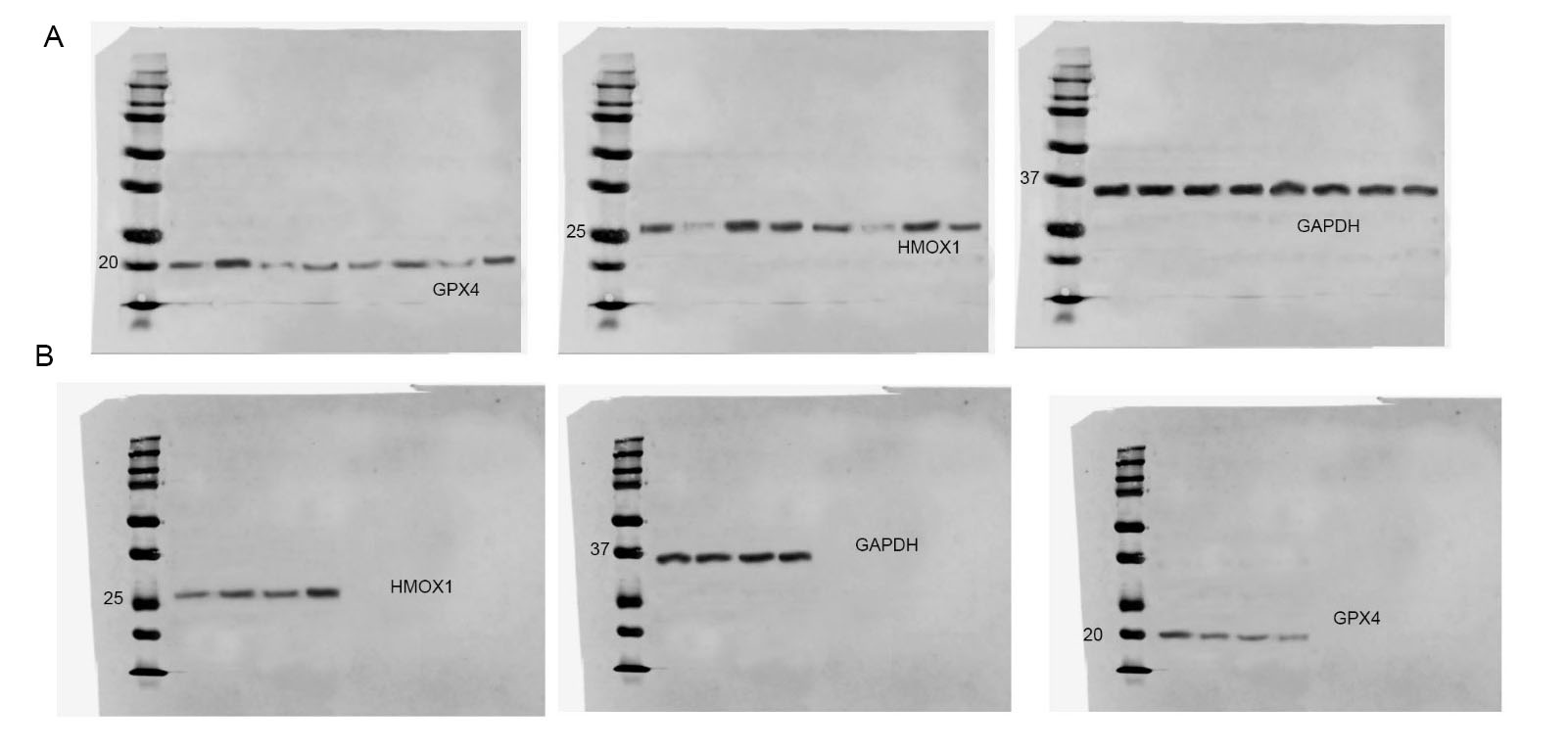


**Supplementary Figure 5. Uncropped western blot images.**

(A-B) Full-length, uncropped blots corresponding to Figures 4 and 6, respectively.

.
